# Live-stream characterization of cadmium-induced cell death using visible CdTe-QDs

**DOI:** 10.1038/s41598-018-31077-2

**Published:** 2018-08-22

**Authors:** Samira Filali, Alain Geloën, Vladimir Lysenko, Fabrice Pirot, Pierre Miossec

**Affiliations:** 1 Immunogenomics and Inflammation Research Unit EA 4130, Department of Immunology and Rheumatology, Edouard Herriot Hospital, Hospices Civils de Lyon, University of Lyon, Lyon, France; 2 Laboratory of Research and Development of Industrial Galenic Pharmacy and Laboratory of Tissue Biology and Therapeutic Engineering UMR-CNRS 5305, Pharmacy Department, FRIPHARM Platform, Edouard Herriot Hospital, Hospices Civils de Lyon, University of Lyon, Lyon, France; 3 CarMeN laboratory, INRA UMR1397, INSERM U1060, INSA Lyon, University of Lyon, Lyon, France; 4 Nanotechnology Institute of Lyon, UMR-CNRS 5270, INSA Lyon, University of Lyon, Lyon, France

## Abstract

Characterization of cell death currently requires the use of indirect markers, which has largely limited the ability to monitor cell death processes inside the cell. Here, we introduce a new method for the characterization of cell death mechanisms using cadmium telluride quantum dots (CdTe-QDs). Using visible CdTe-QDs with mesenchymal cells (e.g. synoviocytes), live-stream imaging allowed for visualization of cadmium-induced cell death, combining characteristics of apoptosis and autophagy. Initially, similar anti-proliferative effect was observed between 10 µg/ml Cd^2+^ and CdTe-QDs at 24 h (cell index/cell density ratio decreased from 0.6 to −16.6, p < 0.05) using techniques that do not require the capacity of CdTe-QDs. Apoptosis was confirmed by the quantification of morphological parameters (reduced surface area, increased cell thickness) and positive labeling with annexin V. Autophagy was confirmed by monodansylcadaverine staining, identifying similar autophagic vacuoles with both Cd^2+^ and CdTe-QD. However, QD imaging allowed for visualization of cadmium elements inside cell structures and their kinetic changes leading to cell death. Cell death characteristics were similar in inflammatory and non-inflammatory environment but were induced up to 4 h earlier in the former. Therefore, live-stream imaging of a visible cytotoxic agent has useful applications not currently possible with indirect methods, including chronological monitoring of cell death.

## Introduction

Characterization of various types of cell death (e.g., apoptosis, necrosis, autophagy) currently requires the use of indirect markers^1,2^ and a combination of different assays (e.g., morphological, immunohistochemical, biochemical, and molecular methods); however, it is not possible to directly investigate the intracellular events leading to cell death. Moreover, these methods are often tedious, time-consuming, and expensive, and do not allow for concomitant identification of the cytotoxic molecules and the process of cell death. The development of each new cytotoxic molecule necessarily requires characterization of cell death. In response to different stimuli induced by these cytotoxic molecules, several types of cell death can be considered: (1) apoptosis, which is characterized by a controlled process of cellular dismantling under non-inflammatory conditions; (2) necrosis, which can be described as an “accidental” process that causes an inflammatory reaction; and (3) autophagic cell death, which involves an increase in autophagy that contributes to cell death under intense metabolic stress^1^. In the present study, we aimed to use a visible compound for simultaneously inducing and characterizing cell death in real-time.

Mesenchymal cells such as macrophages, endothelial cells, and fibroblasts play crucial roles in chronic inflammatory disease by interacting synergistically with activated immune cells recruited to the injury site, leading to their inappropriate survival and accumulation. In the present study, we focused on mesenchymal cells from an inflamed joint (synoviocytes) as a cellular model, which are known to exhibit defects in apoptosis^3^. Homeostasis of metal ions is required in several biological processes, including cell viability. Thus, to overcome this apoptosis resistance, homeostasis of essential metals (e.g., calcium, iron, and zinc) can be disturbed by non-biological elements such cadmium. Indeed, the intra-articular administration of low-dose mineral cadmium (Cd^2+^) was previously shown to induce massive cell death in synoviocytes and protect against joint destruction in an animal model^4,5^. Moreover, Cd^2+^-based quantum dots (QDs) induce cellular cytotoxicity *in vitro*^6–11^. QDs are 1–10-nm-diameter^7^ nanocrystals^12^ that emit intense fluorescence without photobleaching^13,14^. The remarkable properties of QD make them particularly advantageous for monitoring in photonic and electronic imaging, notably owing to their ability of fluorescence emission in the visible wavelength range and their relatively high density, which strongly depends on the size and materials of the particles. In the present study, cadmium telluride quantum dots (CdTe-QDs) were used to directly visualize the events leading to the cell death of synoviocytes in real-time.

## Results

### Satisfactory reproducibility of cell death between Cd^2+^ and CdTe-QDs

The proliferation of synoviocytes treated with QDs was evaluated qualitatively by imaging and quantitatively by cell impedance-based kinetics, enabling a real-time detection of cell death. For both Cd^2+^ and CdTe-QDs, a dose-response assay by imaging determined that a 10 µg/mL concentration induced a complete inhibition of synoviocyte proliferation at 24 hours and a lethal effect at 72 hours (Supplementary Figs 1 and 2). The cellular morphology and proliferation of synoviocytes treated with the negative control non-cytotoxic carbon fluoroxide quantum dots (CFO-QDs) were unchanged. Those indicated that an anti-proliferative effect was similar between Cd^2+^ and CdTe-QDs.

### Imaging of CdTe-QDs-induced apoptosis

**Figure 1:**
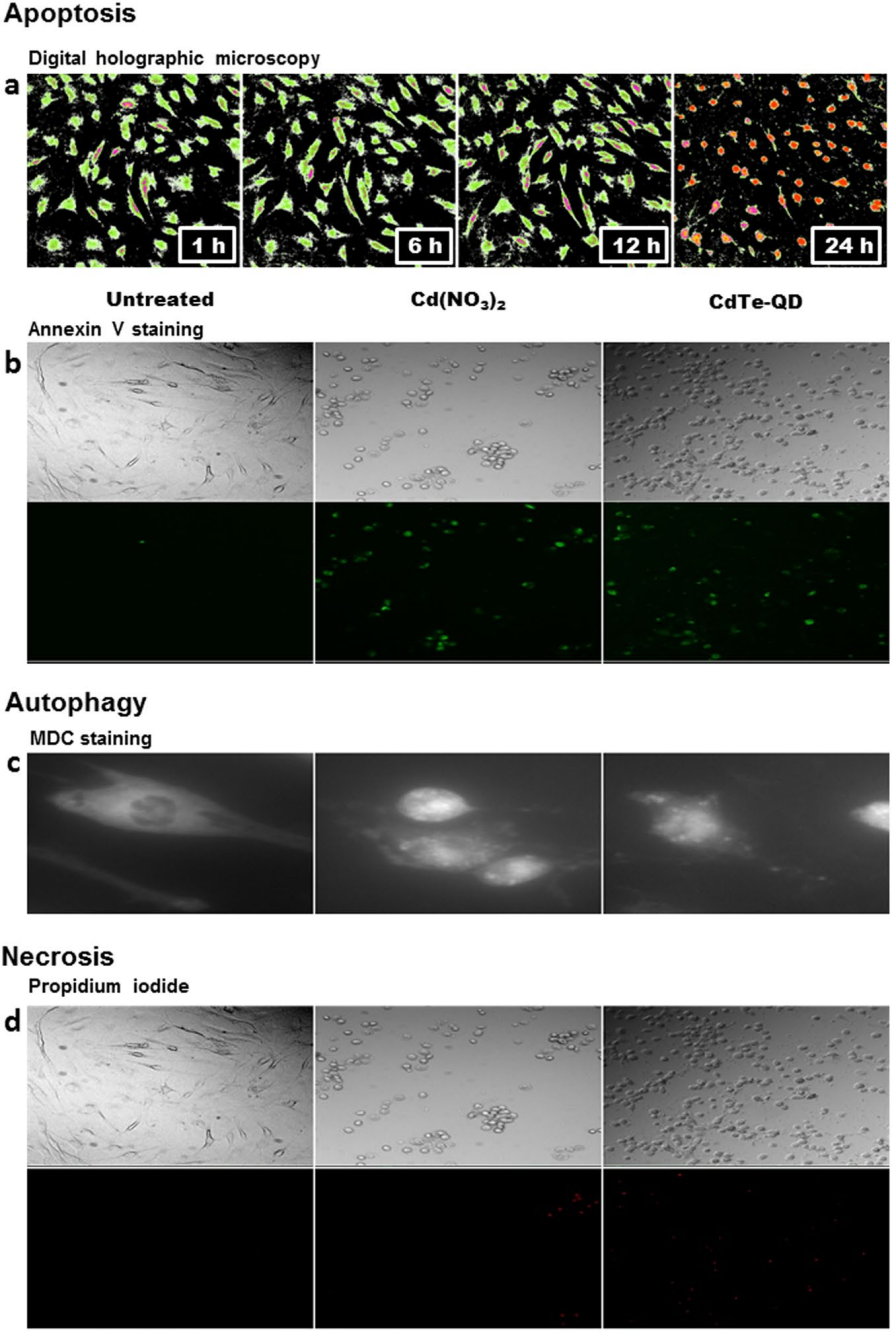
Cell death imaging using conventional methods. (**a**) Digital holograms of synoviocytes treated with CdTe-QDs (λ_ex_: 510 nm) for 1, 6, 12, and 24 hours. Fluorescence micrographs of cells left untreated or treated with Cd(NO_3_)_2_ or CdTe-QDs (10 µg/mL) for 24 hours stained by (**b**) annexin V, (**c**) MDC, and (**d**) propidium iodide.

We characterized apoptosis with different methods (digital holographic microscopy, Annexin V staining, electron microscopy), and the results were compared to those obtained through QD imaging. Digital holographic microscopy showed significant changes in cell morphology at 24 h, including a significant decrease in surface area (mean ± standard error of the mean (SEM): 425 ± 35 µm² vs. 1008 ± 80 µm², *p* < 0.05) and a significant increase in cell thickness (mean ± SEM: 3.90 ± 0.26 µm vs. 1.56 ± 0.10 µm, *p* < 0.05), indicative of apoptosis (Supplementary Video 1, Fig. 1a). Classical Annexin V staining of fixed cells confirmed the induction of apoptosis by revealing phosphatidylserine labeling with both Cd^2+^ and CdTe-QDs (Fig. 1b). Moreover, Hoechst blue staining was used to confirm chromatin condensation (data not shown).

**Figure 2:**
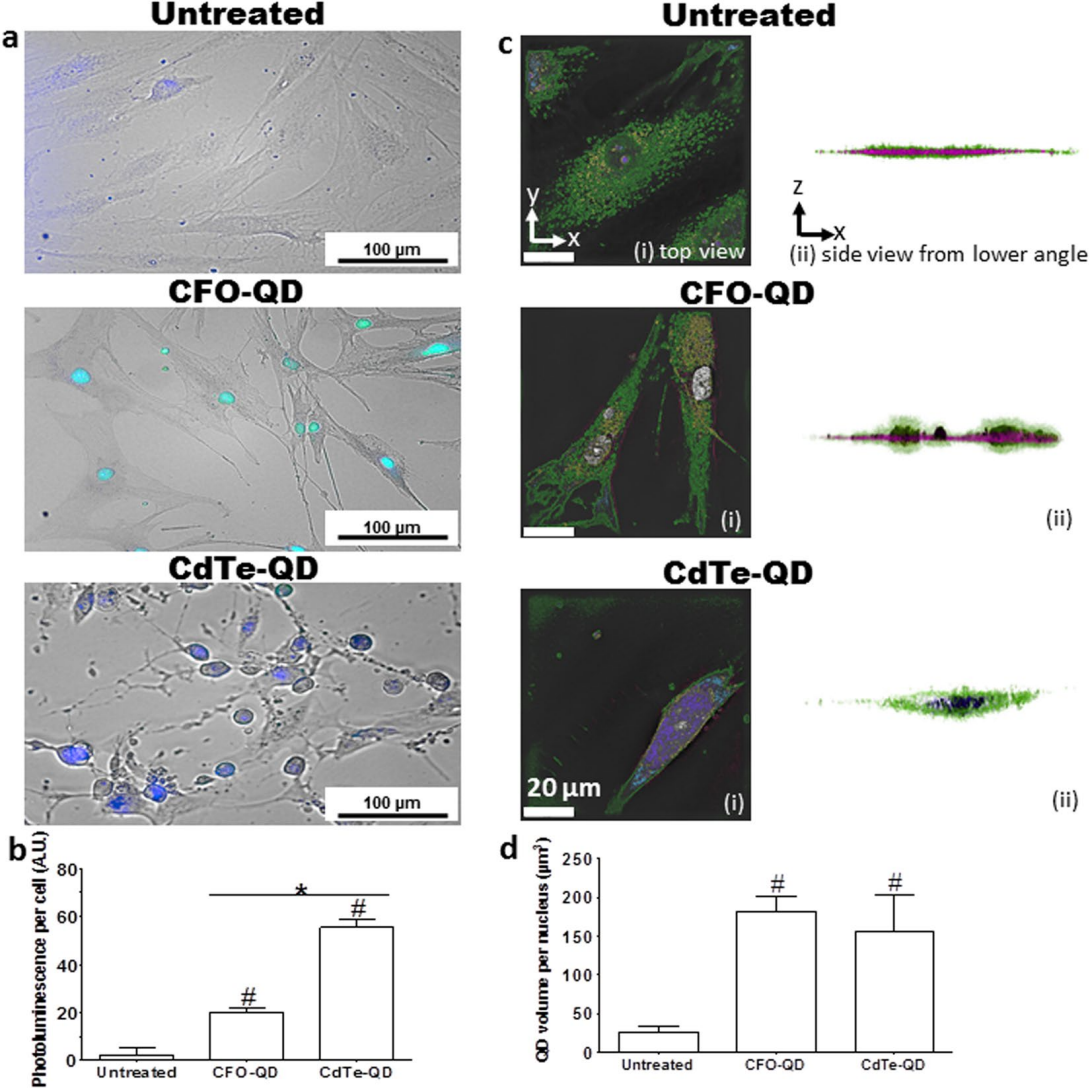
Quantification of cell death by QD imaging. (**a**) Fluorescence micrographs of synoviocytes treated with CFO-QDs and CdTe-QDs compared to untreated cells. (**b**) The photoluminescence of cells (n = 35) is represented by the mean ± SEM. ^#^*p* < 0.01 vs. untreated cells. *p < 0.01 vs. CFO-QDs. (**c**) Three-dimensional holographic and tomographic microscopy images of synoviocytes left untreated or treated with CFO-QDs or CdTe-QDs. (**d**) The QD volume per nucleus is represented by the mean ± SEM. ^#^*p* < 0.01 vs. untreated cells.

**Figure 3:**
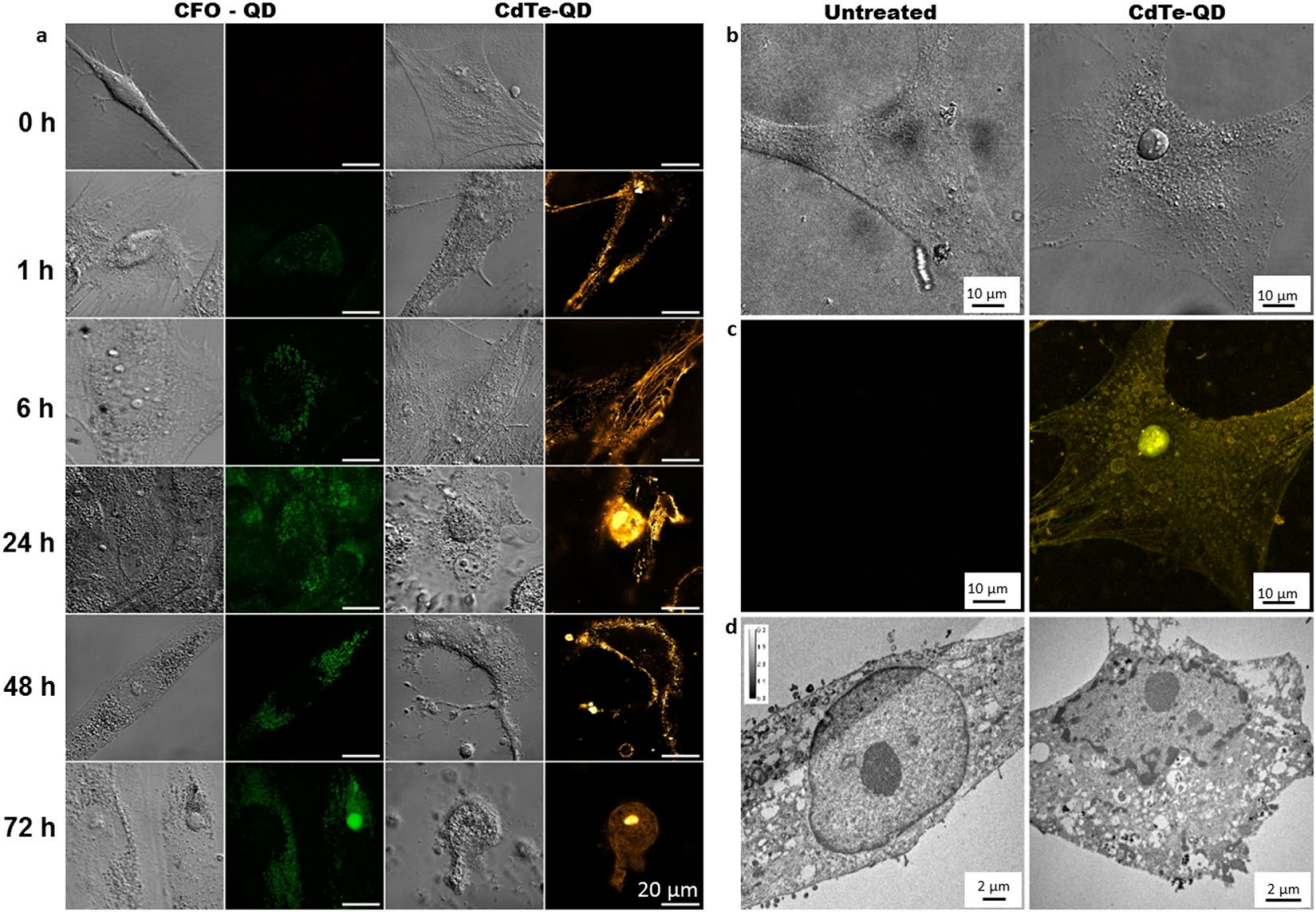
Cell death characterization by QD imaging. (**a**) Confocal micrograph of whole synoviocyte untreated and treated with CFO-QDs and CdTe-QDs (λ_ex_: 670 nm). (**b**,**c**) Confocal micrograph (λ_ex_: 580 nm) and (**d**) transmission electron micrograph of a whole synoviocyte clearly showing evidence of apoptosis and autophagy after treatment with CdTe-QDs.

Qualitative and quantitative analysis of apoptosis by QD imaging was characterized by fluorescence and confocal microscopy. Wide-field merged images obtained by fluorescence microscopy after 24 h clearly demonstrated the rounding and shrinking of cells in the presence of CdTe-QDs, which were not detected with the negative control non-cytotoxic CFO-QDs, and significantly greater fluorescent signals inside synoviocytes were displayed in both groups treated with QDs compared to untreated cells (mean ± SEM: CdTe-QDs: 57.4 ± 3.2 A.U., CFO-QDs: 20.1 ± 1.4 A.U., untreated: 2.2 ± 3.3 A.U., *p* < 0.01, Fig. 2a,b). Powerful magnification images obtained by confocal microscopy demonstrated distinct labeling between CdTe-QDs and CFO-QDs. For CdTe-QDs, filamentous labeling was visible in the cytoplasm for up to 6 h. Surprisingly, naked CdTe-QDs with -COOH functional groups showed the cytoskeletal organization of the cell (Fig. 3a). Beyond 24 h, CdTe-QDs labeling showed condensed chromatin (Fig. 3a). The QD volume per nucleus, measured by tomographic holographic microscopy, was significantly greater compared to that of untreated cells (mean ± SEM; CdTe-QDs: 155 ± 49 µm^3^, CFO-QD: 180 ± 20 µm^3^, untreated: 23 ± 9 µm^3^, *p* < 0.05, Fig. 2c,d). A clear morphological change (Figs 2a and 3a–d, Supplementary Video 2) was observed, with the disappearance of intra-nuclear components in approximately half of the cells and the presence of apoptotic bodies was evident. For CFO-QDs, no characteristic labeling of apoptosis was displayed.

To analyze the mechanism of cell death induced by CdTe-QDs, cytoplasmic CdTe-QDs uptake by direct transfer was observed by electron microscopy. Most of the CdTe-QDs preferentially bound to actin filaments, but not to the microtubules or intermediate filaments. At 24 h, heterochromatin was not clearly distinguishable from the nuclear membrane of untreated cells, while massive condensation of heterochromatin encrusted with CdTe-QDs was observed (Supplementary Fig. 3). These findings demonstrated that the discrimination, development, and monitoring of apoptosis characteristics are feasible using QD imaging.

### Imaging of CdTe-QDs-induced autophagy

The possible contribution of autophagy was concomitantly detected and investigated. Autophagic cell death was characterized with different methods (monodansylcadaverine [MDC] staining, electron microscopy) and compared with the findings obtained through QD imaging. MDC staining showed autophagic flux, with an increase of autophagic vacuoles in the presence of Cd^2+^ and CdTe-QDs (Fig. 1c). Confocal microscopy showed labeling of the cytoplasmic vesicles with CdTe-QDs from the first hour and until 72 h in addition to the filamentous labeling described above. Conversely, CFO-QDs could only label the vesicular regions. The vesicles were heavily loaded with CFO-QDs, and were specifically located in the perinuclear region from the first hour and until 72 h. After 24 h, intense vacuolization of a larger diameter was detected near the nucleus in the presence of CdTe-QDs.

The mechanism of autophagic cell death induced by CdTe-QDs was examined using electron microscopy. CdTe-QDs entered either via clathrin-coated vesicles that were morphologically recognizable by their protein coats, or by caveolin vesicles formed from lipid rafts. After 6 h of exposure, many multi-vesicular bodies containing CdTe-QDs were observed, which were either expelled by exosomes or up taken by auto-phago-lysosomes for their degradation. An increase in autophagic vacuoles containing QDs was also observed (Supplementary Fig. 3). Therefore, monitoring the progress of endocytic vesicle formation was feasible using QD imaging.

### Imaging of lack of evidence of necrosis

Although no signs of necrosis were identified in the previous tests, it was also evaluated using different methods (propidium iodide staining, electron microscopy, and QD imaging). Propidium iodide did not reveal cell death by necrosis (Fig. 1d). Electron microscopy did not reveal cellular or mitochondrial swelling, broken lysosomes, or damage to the cell membrane. These results provide strong arguments for confirming the absence of necrosis.

### Imaging of CdTe-QDs-induced apoptosis and autophagy in an inflammatory environment

**Figure 4:**
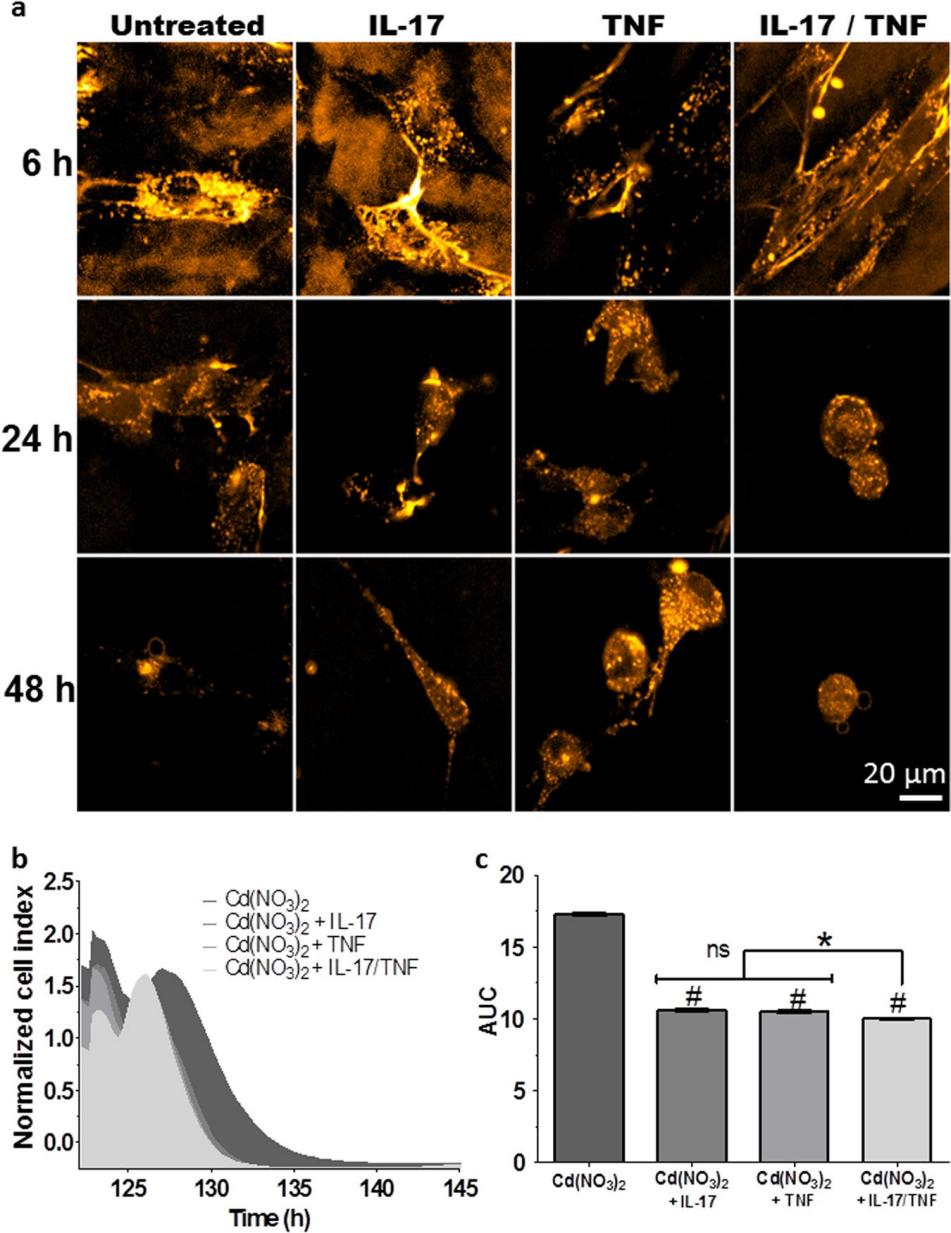
Sensitivity of the new method. (**a**) Confocal micrographs of synoviocytes acquired after 6, 24, and 48 hours exposure to CdTe-QDs (λex: 580 nm, 10 µg/mL) in the presence or absence of inflammatory conditions (IL-17: 50 ng/mL, TNF-α: 1 ng/mL). (**b**) Real-time growth curves of synoviocytes in the presence of Cd(NO_3_)_2_ (1 µg/mL) and inflammatory conditions (IL-17: 50 ng/mL, TNF-α: 1 ng/mL), represented by the normalized cell index (mean values ± SEM, n = 8), as a function of time. (**c**) AUCs with different inflammatory conditions are represented by the mean ± SEM. ^#^*p* < 0.01 vs. untreated cells.

Finally, we evaluated the method in an inflammatory environment, using combinations of the cytokines tumor necrosis factor-α (TNF-α) and interleukin (IL-17) to mimic the pathological conditions of chronic joint diseases^15^. The characterization of cell death was the same as in non-inflammatory conditions, and only the timeline of development changed, with cell death induced up to 4 hours earlier under inflammatory conditions, especially in the presence of both cytokines (Fig. 4a,b). A similar timeline for cell death was observed with Cd^2+^ (mean ± SEM: Cd^2+^: 17.28 ± 0.08 A.U., Cd^2+^  + IL-17: 10.62 ± 0.07 A.U., Cd^2+^  + TNF-α: 10.49 ± 0.07 A.U., Cd^2+^  + IL-17/TNF-α: 10.01 ± 0.06 A.U., *p* < 0.01), and was therefore not due to the use of CdTe-QDs (Fig. 4b,c). The inflammatory environment does not affect the detection of type of cell death but induces the phenomenon early.

## Discussion

Current indirect techniques used to define lethal cell processes do not meet the needs to characterize several types of cell death processes associated with the position of the cytotoxic agent inside the cell over time. Based on a previous study reporting the anti-proliferative and anti-inflammatory effect of Cd^2+^ on synoviocytes^4,5^, we designed a visible Cd^2+^-based new tool, CdTe-QDs, for cell death characterization to define and decompose the different entities involved in the uptake, trafficking, and phenomenon of cell death caused by Cd^2+^ from a structural point of view. In this study, we mainly adopted a semiotic approach (i.e., observation of visual changes between the control and treated cells), which allowed for temporally defining the trajectory of Cd^2+^ in the cell owing to the continuity of the visualization. More specifically, the switch between the different visual realms obtained by these various microscopic techniques revealed the consequence of this element in the cell, especially in the fateful site, the nucleus. This conceptualization also served to highlight the inability of the cell to protect itself from elements such as Cd^2+^ or the Cd^2+^-based particles, harmfully activating the endocytic pathway and leading to cell protection failure. Finally, the use of CdTe-QDs as a Cd^2+^ visualization tool demonstrated the induction of a complex cell death process combining both apoptosis and autophagy, which occurred in a well-defined order.

The effect of Cd^2+^ itself on synoviocytes was demonstrated by first comparing Cd^2+^ and CdTe-QDs, which considers the intrinsic Cd^2+^ ion concentration constituting CdTe-QDs, and then by comparing two QDs consisting of different materials: one composed of Cd^2+^ and the other of carbon. The dose-response curve showed an anti-proliferative effect for the same intrinsic theoretical concentration of Cd^2+^ ions, arguing in favor of a Cd^2+^ effect. The absence of an anti-proliferative effect for the QDs composed of carbon suggested that cell death was induced by Cd^2+^ and not by a so-called “cargo effect” of QDs, indicating the critical need of a control QD. In other words, CdTe-QDs monitoring would be representative of the trajectory and effect of Cd^2+^. Furthermore, the impedance system helped to define the timing of cell death and to deduce the rate of the process. Therefore, the anti-proliferative effect was reproducible between Cd^2+^ and CdTe-QDs, but the effect was postponed according to the size of the nanoparticles. This shift is probably related to the time it took for the compound to enter the cells. Once inside, Cd^2+^ caused faster cell death than the CdTe-QDs, which required more time to reach the nucleus. The amount of intrinsic Cd^2+^ ions required per cell to achieve a similar cell death rate compared to CdTe-QDs was estimated at 100 pg/cell using the same impedance system. Thus, the monitoring by QD revealed two types of stimuli in these resistant synoviocytes: apoptosis and autophagy.

By comparing our new QD imaging method to conventional methods for the ability to characterize apoptosis, we demonstrated multiple improvements regarding the experimental duration, possibility of using live cells, availability of equipment, lower costs, and the exceptional image clarity (Supplementary Table 2). Although digital holographic microscopy was performed on living cells without requiring additional reagents and ensuring the quantification of morphological parameters, this method is accompanied by an important limitation in that it is not possible to test several conditions simultaneously. Annexin V staining, which is performed on fixed cells, is seen as the reference technique for apoptosis detection. However, since Annexin V denatures the cells after staining, this method precludes the examination of kinetics in the same cells. Therefore, QD imaging is convenient to implement and is reproducible for conducting both the qualitative and quantitative analysis of apoptosis. After 24 h of exposure, the presence of the toxic element inside the cells was confirmed owing to wide-field merged images obtained by fluorescence microscopy. Different spatiotemporal localizations were observed for CdTe-QDs compared to those of CFO-QDs. These localizations chronologically determined the onset of different signals leading to cell death. Surprisingly, after direct integration during the first hour, CdTe-QDs with their -COOH terminal function were first trapped by the actin microfilaments, which could be attracted by the positive ends in the periphery and were distributed throughout the cytoplasm after 6 h of exposure. To our knowledge, this is the first demonstration of the successful labeling of actin microfilaments by naked QDs to date. Interestingly, the CFO-QDs, which have the same -COOH terminal function, did not enter the cell via a non-endocytic route, and therefore had no such affinity for actin microfilaments. These results suggest that it is its metallic nature contributing to the affinity of QD for actin microfilaments, which in turn favors a complexation reaction or precipitation. Cd^2+^ was transported to the nucleus with preferential attraction for heterochromatin, which triggered the process of cell death by apoptosis. The initiation of cell death appeared once the CdTe-QDs reached the nucleus as observed after 24 h. This is correlated with the results obtained with the cell impedance system where normalized cell index changes were observed from the 15^th^ and 25^th^ hour. Apart from this study, the morphological appearance of apoptosis was confirmed with a known cytotoxic agent inducing apoptosis (i.e., bortezomib) in synoviocytes^16^, inducing the same apoptotic changes. The changes induced by apoptosis were investigated on another cell type (panc cell line) and another agent known to induce apoptosis (i.e., cyclosporin)^17^ compared to CdTe-QDs, showing the same apoptotic changes between CdTe-QDs and ciclosporin (Supplementary Fig. 4). The non-endocytic pathway was predominant and governed the apoptosis induced by Cd^2+^. Besides allowing for clear characterization of apoptosis, QD imaging further provided information to explain the interaction of Cd^2+^ with the elements inside the cell and to understand the pathway leading to apoptosis.

As for the detection of apoptosis, this new QD imaging method demonstrated various improvements for autophagy characterization compared to conventional methods. The visualization of autophagic vacuoles was more clearly displayed with QD imaging than by the conventional method of MDC staining. This was used to evaluate the induction of autophagy through the accumulation of autophagic vacuoles without reflecting autophagosomal maturation and endocytic pathway^18–21^. Furthermore, the non-destructive nature of QD imaging allowed for visualization of vesicle enlargement over the course of the experiment, which was impossible with MDC. The endocytic pathway by caveolin and clathrin ensured detoxification of the cell by expelling Cd^2+^ via the exosomes or its removal through lysosomal degradation, which oriented the pathway toward autophagy. Once this autophagy became ineffective, these events contributed to cell death. This latter phenomenon was highlighted by the significant presence of large autophagic vacuoles containing CdTe-QDs as early as 24 h with an increase of their sizes up to 48 h. From this time up to 72 h, the apoptotic changes (the net rounding of cells and the formation of apoptotic bodies) were concomitantly highlighted. For summary, the kinetics of apoptosis and autophagy signs were as follows: 1 h: uptake by the non-endocytic and endocytic pathway; 6 h: distribution throughout the cytoplasm via actin microfilaments and multivesicular body formation; 24 h: condensation of chromatin and presence of autophagic vacuoles; 48 h–72 h: rounding of cells, increase in size of autophagic vacuoles and formation of apoptotic bodies. The morphological appearance of autophagic vacuoles was confirmed with a known autophagy inducing agent (i.e. metformin)^22^, showing the same autophagic signs after 1 hour of CdTe-QDs exposure without induction of apoptosis (Supplementary Fig. 4). At the molecular level (which was not the focus of our study), three previous studies showed a strong caspase involvement in the apoptotic process of CdTe-QDs in HUVEC and CdSe/ZnS-QD cells with terminal function -COOH in mesenchymal cells by using a caspase inhibitor zVAD.fmk. One of them also suggests the involvement of autophagy in cell death. In mesenchymal cells, inhibition of apoptosis with zVAD.fmk has been shown neither to affect the level of autophagy nor to prevent cell death^23–25^. Thus, QD imaging not only permitted clear characterization of autophagy, but further allowed for tracing of the evolution of the phenomenon of autophagy ranging from cellular protection to ultimate cell death.

The phenomenon of necrotic cell death is mainly characterized by an increase in cell volume, swelling of the organelles, and an increase in the permeability of the plasma membrane of cells. These events contribute to disruption of the plasma membrane, resulting in the release of the cellular contents into the surrounding medium, which triggers a strong inflammatory reaction. Here, the imbalance of homeostasis of the essential metals did not trigger the events that are characteristic of necrosis. The lack of necrosis induction is important and crucial information that highlights the potential for the development of new Cd-based cytotoxic therapies in the context of chronic inflammatory diseases.

Toward this end, we further evaluated the sensitivity of the method under inflammatory conditions. Apoptosis and autophagy were induced by CdTe-QDs to the same extent as observed in the non-inflammatory condition, although they were triggered earlier. This relatively early induction of cell death in an inflammatory environment could be explained by the changes in cell morphology induced by cytokines (increased numbers of pseudopodia), which would allow for increased Cd^2+^ and CdTe-QDs uptake. In fact, the numerous pseudopodia on the cell surface increase the collecting surface of elements. Indeed, a previous study demonstrated that cytokines induced morphological changes in microglial cells and astrocytes, including time-dependent cytoskeletal changes, and increased the number of filopodia in microglial cells^26^. However, few studies have examined the improved uptake of compounds under inflammatory conditions; thus, this issue needs further clarification in more detailed studies. The QD imaging method clearly highlighted that the difference observed between the inflammatory and non-inflammatory conditions had an impact on the initiation of the anti-proliferative effects without affecting any of the processes occurring inside the cell. Indeed, the clear increase of fluorescence and the morphological changes characteristic of cell death perceived early in the inflammatory condition indicated that the phenomenon had indeed occurred. These kinetic observations on cell death were also found with Cd^2+^ in the inflammatory condition using the same impedance system.

## Conclusion

In conclusion, as compared to conventional methods, the use of CdTe-QDs enabled the direct and simultaneous visualization of different modes of Cd-induced cell death. Live-stream characterization of cell death showed that CdTe-QDs uptake by both the endocytic and non-endocytic pathways simultaneously led to apoptosis (with chromatin condensation and apoptotic bodies detectable) and autophagy (based on observation of autophagic vacuoles containing CdTe-QDs). This newly developed live-stream QD imaging method is a promising tool for the characterization and monitoring of the type of cell death but not to quantify the general toxicity of CdTe-QDs. This method has potentially useful applications, including monitoring of kinetic changes associated with cell death processes on other types of cells, through investigation of the homeostasis imbalance of the essential metals by other metals or with a combination of different metals on various type of cells. In the future, for the specific case of Cd^2+^, CdTe-QDs may be used in animal models to investigate the spread of Cd^2+^ and to define the cytotoxic effects of Cd^2+^-based QDs in different cell types toward clinical application.

## Methods

### Materials

Cadmium nitrate solutions were obtained from the geology laboratory of the University of Lyon (UMR 5276). Carbon fluoroxide nanoparticles were synthesized and characterized as described by Lysenko V. *et al*. at the Institute of Nanotechnology of Lyon^27,28^. Three sizes (1.53 nm, 3.24 nm, 3.99 nm) of hydrophilic CdTe-QDs (coated with -COOH groups) in powder form emitting at three different wavelengths (green, λ_ex_: 510 nm; orange, λ_ex_: 580 nm; red, λ_ex:_ 670 nm) were purchased from PlasmaChem GmBH (Berlin, Germany; Supplementary Table 1). CdTe-QDs were reconstituted with pyrogen-free water-for-injection (Lavoisier^®^, Paris, France) and then filtered through a 0.20 µm polytetrafluoroethylene filter (MILLEX^®^, Merck Millipore, Darmstadt, Germany). Non-cytotoxic carbon fluoroxide QDs (CFO-QDs), which have similar structure to CdTe-QDs in terms of form, hydrophilicity, core size, visible emission and equal photoluminescence, were used as negative control (Supplementary Table 1). Water was prepared using a Millipore Direct-Q 8 purification system for all other purposes.

Dulbecco’s Modified Eagle Medium (DMEM), Trypsin – Versene (EDTA; Eurobio, Les Ulis, France) was supplemented with 10% (w/v) fetal bovine serum (FBS; Gibco, Grand Island, NY, USA), 2% (w/v) penicillin/streptomycin (Eurobio), 1% (w/v) L-glutamine (Eurobio), amphotericin B (Eurobio), plasmocyn prophylactic (InvivoGen, Toulouse, France) and then filtered through a 0.20 µm polytetrafluoroethylene filter (Millex, Merck Millipore). IL-17A was provided by Dendritics (Lyon, France) and TNF by R&D systems (Lille, France). The Annexin-fluos kit was purchased from Roche Molecular Biochemicals (Mannheim, Germany).

### Cell culture

Rheumatoid arthritis - fibroblast-like synoviocytes were grown from synovial tissue of three patients undergoing joint surgery, who fulfilled the American College of Rheumatology criteria for rheumatoid arthritis. Each individual signed an informed consent form and the protocol was approved by the Lyon teaching Hospitals review board (number AC-2016-27-29) according to French Public Health laws (art R1243-57, art L1121-1-1, art L 1121-1-2). All methods were performed in accordance with the relevant guidelines and regulations. Synovial tissue was minced into small pieces which were allowed to adhere to 6-well plates in supplemented DMEM, as described above. RA-FLS grew out of the tissue and colonized the plastic dishes until reaching confluence. Synoviocytes were then trypsinized and grown in 75 cm^2^ cell culture flasks in a humidified atmosphere (5% v/v CO_2_) at 37 °C. Growth medium was replaced twice a week, and when the cells were confluent they were trypsinized and transferred in two 75 cm^2^ cell culture flasks for continued growth. The cells used in this study were recovered from between the 4^th^ and 9^th^ cell passage.

### Dose-response assay

Change of cellular morphology was observed using an inverted optical microscope (Zeiss Axiovert 200 M fluorescent microscope, Carl Zeiss Microscopy, Oberkochen, Germany) with a magnification of x40. Synoviocytes were incubated in the presence of increasing concentrations of CFO-QDs (negative control; 0.25, 2.5, 25, 250 µg/mL), of Cd(NO_3_)_2_ (positive control; 0.01, 0.1, 1, 10 µg/mL) and three sizes of CdTe-QDs (0.01, 0.1, 1, 10 µg/mL). The desirable characteristics were rounding, cell detachment, loss of adhesion, and cytoplasmic retraction.

### Cytotoxicity with real-time and label-free monitoring

The effects of QDs on cell viability were examined using the xCELLigence real-time cell analysis system (Roche Diagnostics GmbH, Mannheim, Germany). The change over time in cellular proliferation was determined by impedance of RA-FLS cells grown in under inflammatory conditions (IL-17 or TNF alone or in combination) or not, and at different seeding densities (7,500, 15,000, 22,500, and 30,000 cells/cm²) in E-plates 96 (Roche Diagnostics GmbH). Three parameters are taken into account by the RTCA software to determine normalized cell index (NCI): cell number, cell area, and cell adhesion strength. CI is a dimensionless parameter and is derived, according to the following equation:

$${\rm{CI}}=\mathop{\max }\limits_{1=1,\,\ldots ,{\rm{N}}}(\frac{{\rm{Rcell}}({{\rm{f}}}_{1})}{{\rm{Rb}}({{\rm{f}}}_{1})}-1)$$

where Rb(f) is the frequency-dependent electrode resistance (a component of impedance) without cells and Rcell(f) with cells, and N is the number of the frequency points at which the impedance is measured. Cell proliferation leads to a higher CI value because of larger Rcell(f), while cytotoxicity induces rounding-up and detachment of cells leading to a decreased CI value. The NCI was measured every five minutes for the first 4 hours and then every 15 minutes from 5 to 87 hours. After 72 hours of proliferation in DMEM, the following conditions were tested: DMEM alone, and with either Cd(NO_3_)_2_, CFO-QDs, or CdTe-QDs (λ_exc_ = 510, λ_exc_ = 580, or λ_exc_ = 670). The NCI was also used to calculate the area under curve (AUC) at each timepoint.

### Enzyme-linked immunosorbent assays (ELISA)

IL-6 production was measured using commercially available ELISA kit, according to the manufacturer’s instructions (R&D Systems, Lille, France).

### Fluorescence microscopy

The quantification of fluorescence of the untreated and labeled cells was determined using a fluorescence microscope (Cytation 3 Cell Imaging Multi-Mode Reader, Biotek Instruments Inc, Winooski, VT, USA) with an excitation of 490 nm and an emission 525 nm.

### Confocal scanning microscopy

High resolution imaging was performed using a confocal laser scanning Zeiss LSM 78-NLO microscope (Carl Zeiss Microscopy) equipped with 100x objective (with an oil-immersion objective (alpha “Plan-Apochromat” 100X/1.46 Oil DIC) with laser diode 405 nm 30 mW, 561 nm 20 mW) and a photomultiplier tube (PMT) detector (360 to 800 nm), and a 34-channel spectral detector (470 to 670 nm). Cells were seeded into the wells of two-compartment Nunc culture chambers (LabTek^®^ II non-separable, Dutscher scientific, Brumath, France) at a density of 50 000 cells/cm^2^. The excitation wavelength was 405 nm and the laser power was 5%. Imaging was carried out using QD 670 at 10 µg/mL, and detection in spectral mode. The master gain remained the same for all sessions. The cells were incubated for 1, 6, 24, 48, and 72 hours, and then washed twice with PBS to remove any non-specifically adsorbed QD. The PMT gain was adjusted at each acquisition to obtain the best image quality with good contrast. Stacks of optical sections were collected by optical z sectioning (z step = 0.4 µm). Auto fluorescence of FLS (ranging from 400 to 600 nm) was deconvoluted using spectral unmixing algorithms (ZEN 2012, Carl Zeiss Microscopy). Images were analyzed using ImageJ software (National Institutes of Health, Bethesda, MD, USA). Fluorescence calculations were performed using ZEN 2012 software (Carl Zeiss Microscopy). Three-dimensional images of synoviocytes were rendered using ICY software (http://icy.bioimageanalysis.org) and the 3D Rotation plugin.

### Electron microscopy

After fixing cells as previously described^20^, images were acquired using a JEM Jeol 1400 transmission electron microscope (Jeol Ltd., Tokyo, Japan) operated at 80 kV coupled to a digital camera (Orius 600, Gatan Inc., Pleasanton, CA, USA) and processed using Digital Micrograph^®^ software (Gatan Inc.).

### Digital holographic microscopy

The quantitative morphological parameters (cell area, volume, and thickness) were defined by analysis of cellular kinetics over 25 hours by HoloMonitor^®^ M4 (Phase Holographic Imaging AB, Lund, Sweden) with and without smallest CdTe-QDs. The digital holographs were recorded every five minutes.

### Living cell tomography

The visualization of the 3D morphologies of synoviocytes and localization of the QDs were performed by interferometric detection using tomographic holographic 3D microscopy Nanolive^®^ (3D Cell Explorer, Lausanne, Switzerland), and processed using STEVE^®^ software (3D Cell Explorer). Each color in the figures represents a different refractive index range. In addition, this technique allowed to quantify the volume of QDs in synoviocytes after 24 hours of exposure.

### Annexin V-fluorescein isothiocyanate conjugated with propidium iodide staining

After exposure to Cd^2+^ or CdTe-QDs, synoviocytes apoptosis was quantified by Annexin-V-FLUOS Staining kit according to the manufacturer’s instructions (Roche Molecular Biochemicals, Mannheim, Germany), using Zeiss Axiovert 200 M fluorescent microscope (Carl Zeiss Microscopy; magnification x40).

### Monodansylcadaverine (MDC) staining

After exposure to Cd^2+^ or CdTe-QDs, autophagic vacuoles in synoviocytes were labeled with 0.05 mmol/L MDC in PBS at 37 °C for 10 min. The cells were then washed three times with PBS and then observed under a Zeiss Axiovert 200 M fluorescent microscope (Carl Zeiss Microscopy; magnification x40).

### Statistical analysis

All experiments were repeated three times. Results are presented as mean ± standard error of the mean (SEM). All statistical tests were performed by one-way analysis of variance (ANOVA) followed by Dunnett HSD post hoc using Graphpad version 5.0 for Windows (GraphPad Software, La Jolla, California, USA). P values < 0.05 were considered as significant.

## Electronic supplementary material

Supplementary information

Supplement video
