# Supplementary material for: Live-stream characterization of cadmium-induced cell death using visible CdTe-QDs
Source: Sci Rep. 2018 Aug 22;8:12614. doi: 10.1038/s41598-018-31077-2 (PMC6105671; doi:10.1038/s41598-018-31077-2)
Supplement: Supplementary file 1 — Supplementary information [file 41598_2018_31077_MOESM1_ESM.pdf]

Live-stream characterization of cadmium-induced cell death using visible CdTe-QDs

Samira Filali[1,2*], Alain Geloën[3], Vladimir Lysenko[4], Fabrice Pirot[2], Pierre Miossec[1]

[1] Immunogenomics and Inflammation Research Unit EA 4130, Department of Immunology and Rheumatology, University of Lyon, Lyon, France

[2] Laboratory of Research and Development of Industrial Galenic Pharmacy and laboratory of tissue biology and therapeutic engineering UMR-CNRS 5305, Plateform FRIPHARM, University of Lyon, Lyon, France

[3] CarMeN laboratory, INRA UMR1397, INSERM U1060, INSA Lyon, University of Lyon, Lyon, France

[4] Nanotechnology Institute of Lyon, UMR-CNRS 5270, INSA Lyon, University of Lyon, Lyon, France

Supplementary Figure 1: Dose-response assay of the anti-proliferative effects of $Cd(NO_3)_2$, CdTe-QDs, and CFO-QDs

Changes in synoviocytes morphology were observed after 72 hours exposure to CFO-QDs (0.25, 2.5, 25, or 250 µg/mL), $Cd(NO_3)_2$, and CdTe-QDs (Cd: 0.01, 0.1, 1, or 10 µg/mL).

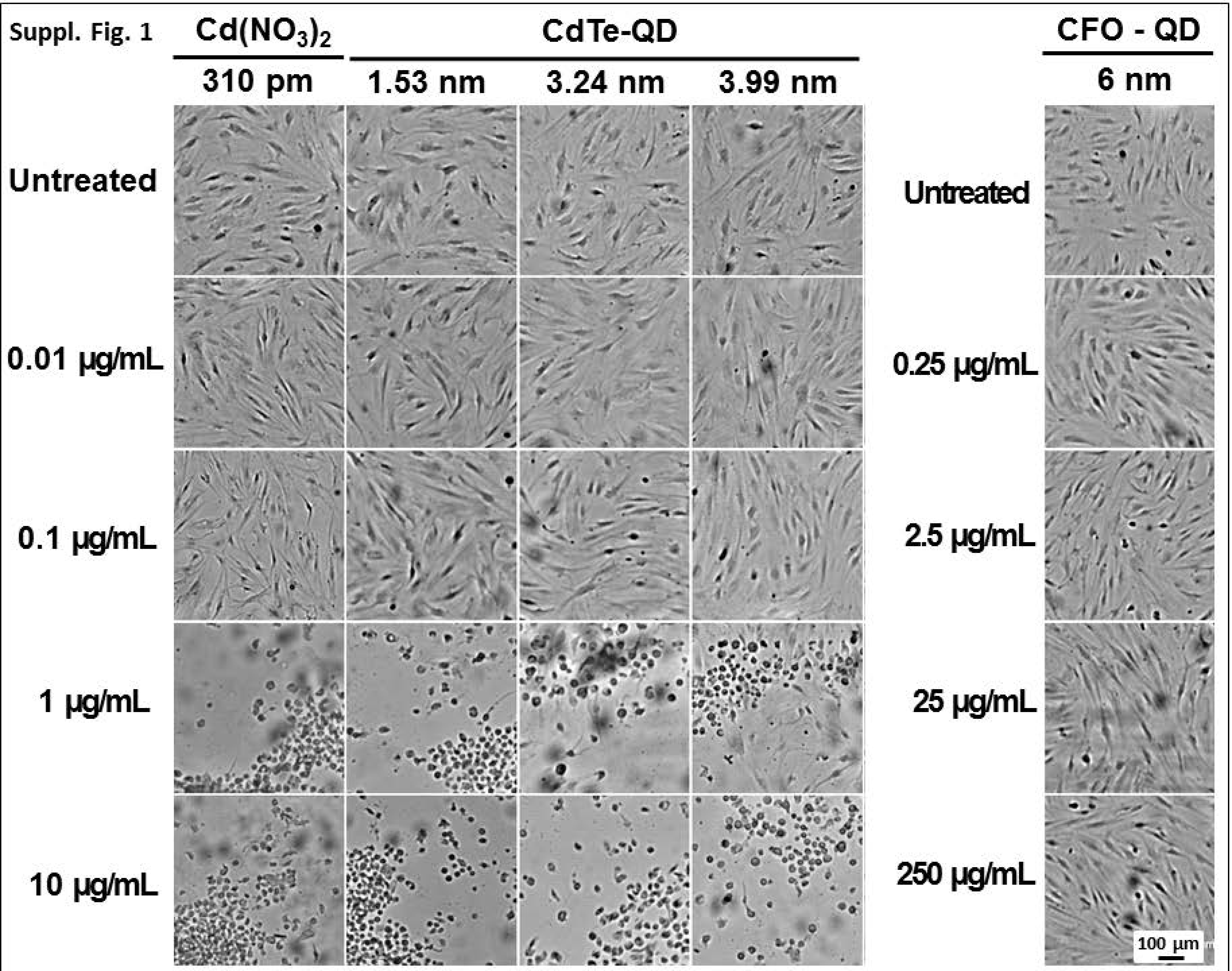

Suppl. Fig. 1
Cd(NO3)2
CdTe-QD
CFO - QD
310 pm
1.53 nm
3.24 nm
3.99 nm
6 nm
Untreated
0.01 µg/mL
0.1 µg/mL
1 µg/mL
10 µg/mL
Untreated
0.25 µg/mL
2.5 µg/mL
25 µg/mL
250 µg/mL
100 µm

Supplementary Figure 2: Quantification of the anti-proliferative effects induced by Cd^{2+} and CdTe-QDs on synoviocyte (a) Real-time growth curves of synoviocytes with different treatments (CFO-QD, $Cd(NO_3)_2$, CdTe-QD), represented by the normalized cell index (mean values ± SEM, n = 8), as a function of time. (b) The positive and negative slopes of cell index correspond to the cell proliferation and cell death kinetics, respectively. #, $p < 0.01$ vs. untreated cells. (c) Cell death kinetics in the presence of $Cd(NO_3)_2$ and CdTe-QD were identical at 100 pg Cd per cell. Data were fitted to a polynomial model (R = 0.999).

Suppl. Fig. 2

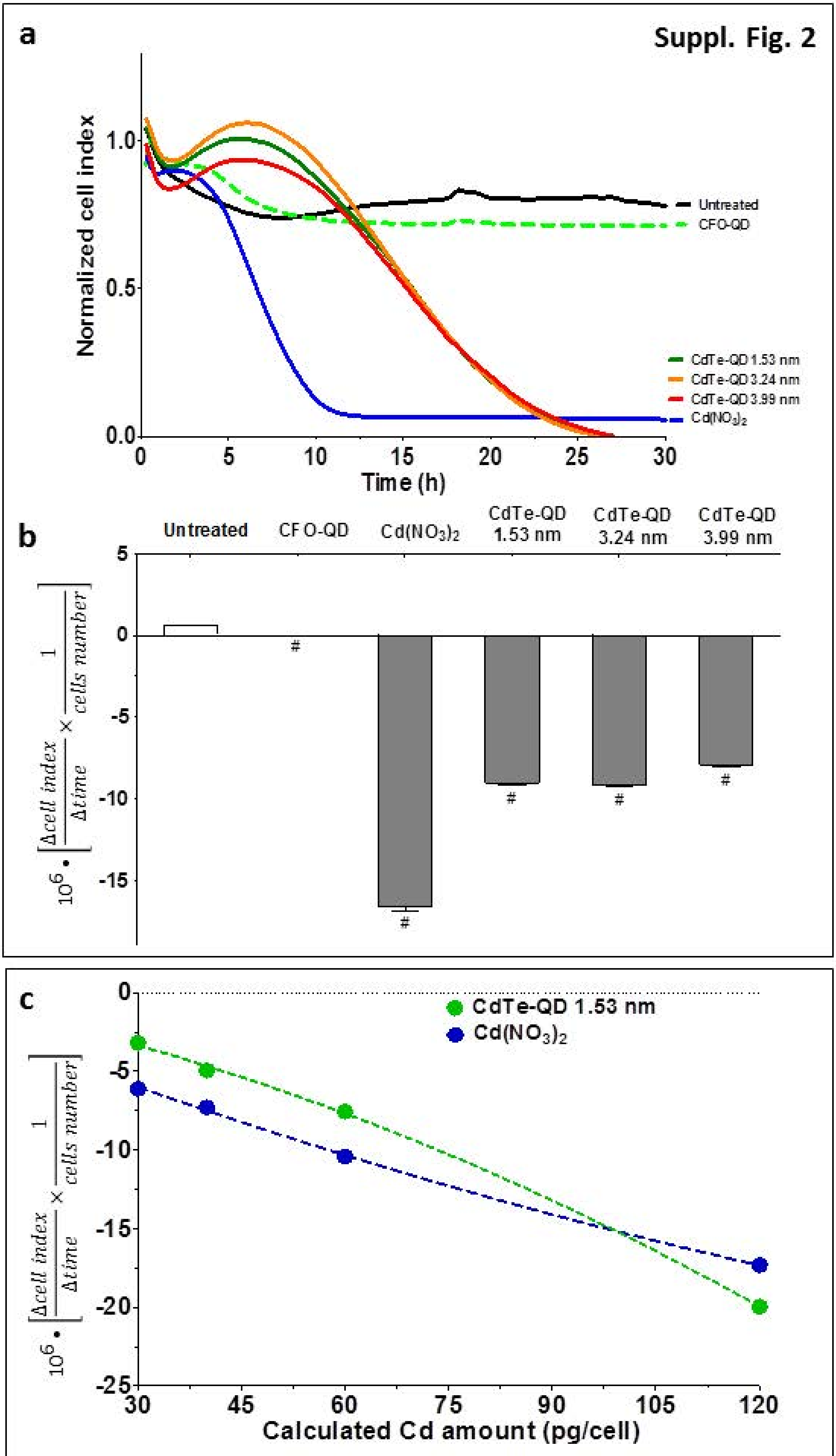
a
Normalized cell index
Time (h)
Untreated
CFO-QD
CdTe-QD 1.53 nm
CdTe-QD 3.24 nm
CdTe-QD 3.99 nm
$Cd(NO_3)_2$
b
Untreated
CFO-QD
$Cd(NO_3)_2$
CdTe-QD 1.53 nm
CdTe-QD 3.24 nm
CdTe-QD 3.99 nm
$10^6 \cdot \left[\frac{\Delta cell\ index}{\Delta time} \times \frac{1}{cells\ number}\right]$
#
c
CdTe-QD 1.53 nm
$Cd(NO_3)_2$
$10^6 \cdot \left[\frac{\Delta cell\ index}{\Delta time} \times \frac{1}{cells\ number}\right]$
Calculated Cd amount (pg/cell)

Supplementary Figure 3: Cd^{2+} uptake in synoviocytes through the endocytic and non-endocytic pathways

Transmission electron micrographs of synoviocytes exhibiting (a) CdTe-QD uptake by direct transfer through the membrane and by vesicle transport; (b) CdTe-QDs linked to actin microfilaments, and the size of the CdTe-QDs. Non-endocytic pathways: (c, d) membrane; (e, f) actin filaments, microtubules, and intermediate filaments; (g, h) euchromatin and heterochromatin; (i, j) the nucleus. Endocytic pathways: (k, l) caveolin vesicles; (m, n) clathrin-coated vesicles; (o, p) endosomes; (q, r) multi-vesicular bodies; (s, t) lysosomes; (u, v) auto-phagolysosomes; (w, x) exosomes; (y, z) autophagic vacuoles.

Suppl. Fig. 3

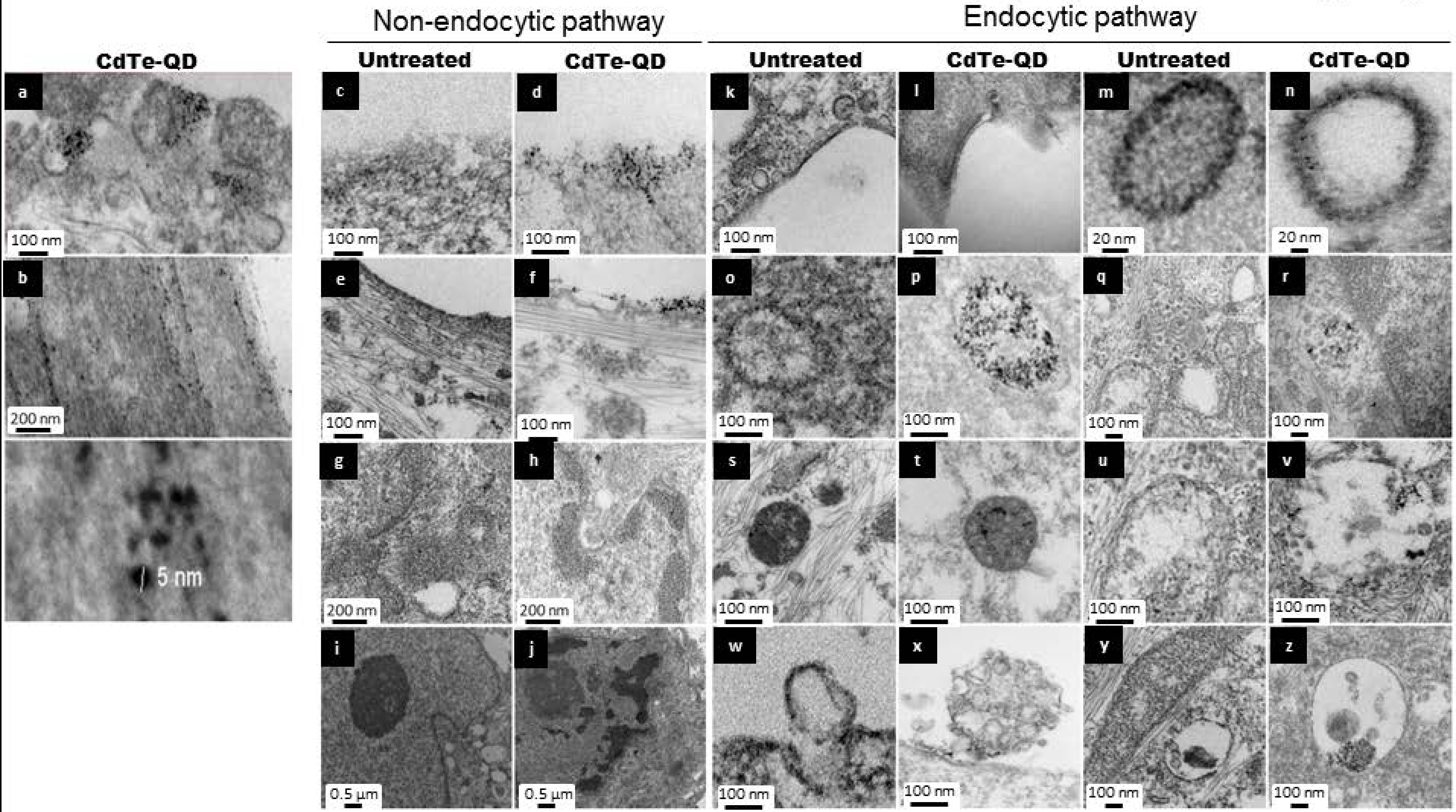

Non-endocytic pathway
Endocytic pathway
CdTe-QD
Untreated
CdTe-QD
Untreated
CdTe-QD
Untreated
CdTe-QD
a
b
c
d
e
f
g
h
i
j
k
l
m
n
o
p
q
r
s
t
u
v
w
x
y
z
100 nm
200 nm
5 nm
20 nm
0.5 µm

Supplementary Figure 4: Confirmation of apoptotic and autophagic labeling and apoptotic-induced morphological change using apoptotic and autophagic inducers

(a) Confocal micrographs of synoviocytes acquired after 24 hours exposure to an apoptosis inducer (bortezomib, 10 nM) or CdTe-QDs (10 µg/ml) or bortezomib (10 nM) then 1 hour exposure with CdTe-QDs (10 µg/ml). (b) Confocal micrographs of synoviocytes acquired after 24 hours exposure to an autophagy inducer (metformin, 10 mM) or metformin (10 mM) then 1 hour exposure with CdTe-QDs (10 µg/ml). (c) Holographic and tomographic microscopy images of panc cells line treated with an apoptosis inducer (cyclosporin, 27 mM) or CdTe-QDs (10 µg/ml).

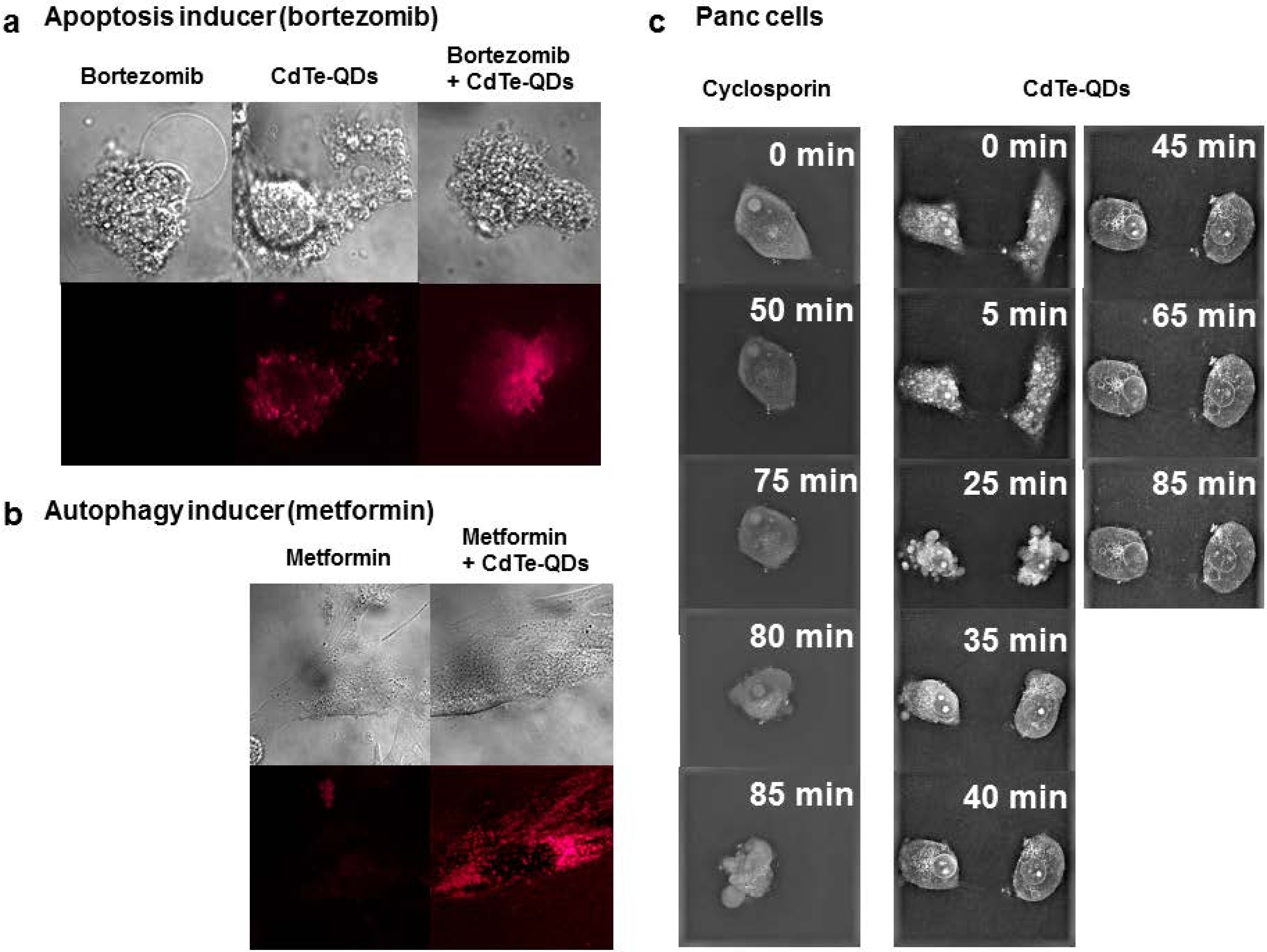

a
Apoptosis inducer (bortezomib)
Bortezomib
CdTe-QDs
Bortezomib + CdTe-QDs
b
Autophagy inducer (metformin)
Metformin
Metformin + CdTe-QDs
c
Panc cells
Cyclosporin
CdTe-QDs
0 min
50 min
75 min
80 min
85 min
0 min
5 min
25 min
35 min
40 min
45 min
65 min
85 min

Supplementary table 1 : Physicochemical characterization of CFO-QD and CdTe-QD.

Physicochemical characteristic	**Carbon fluoroxide (CFO-QD)**	**Cadmium telluride (CdTe-QD)**		
**Analog**				
**Shape**	Spherical	Spherical		
**Size (nm)**	6	1.53	3.24	3.99
**Surface chemical**	- COOH	- COOH		
**Average molecular weight (g/mol)**	2500	3200	67 000	124 000
**Emission wavelength (nm)**	Visible domain	Visible domain (510 - 580 – 670 nm)		
**Opposite**				
**Composition**	**Carbon fluoroxide**	**Atomic ratio Cd : Te [Cd : Te]**		
		[3 : 1]	[5 : 1]	[6 : 1]
**Cell effect**	Non-cytotoxic	Cytotoxic		

Supplementary table 2 : Technical and economic comparison of the different methods for characterizing apoptosis and autophagy.

	Experimental duration	Possibility of using live cells,	Number of samples	Availability of equipment,	Costs	Image quality	Characterization
QD imaging (confocal or fluorescence microscopy)	kinetics	Living cells	several conditions (unrestricted)	available	330 tests (87 euros)	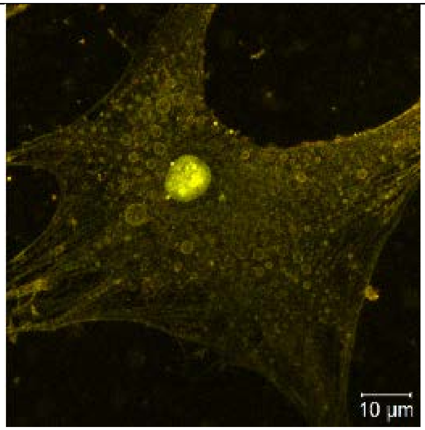	- Uptake<br>- Trafficking<br>- Apoptosis<br>- Autophagy
Digital holographic microscopy<br><br>(Holomonitor M4) low resolution	kinetics	Living cells	1 condition per test	instrument unusual	Price of equipment (20,000 euros)	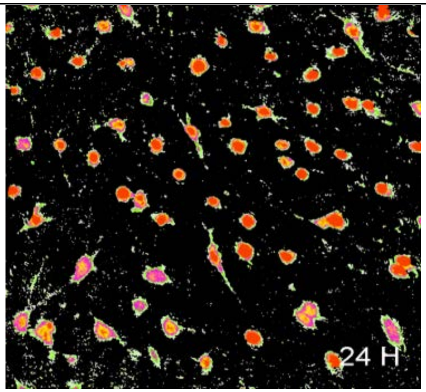	- Apoptosis<br>- Necrosis

Holographic and tomographic microscopy (Nanolive) high resolution	kinetics	Living cells	1 condition per test	instrument unusual	Price of equipment (20,000 to 40,000 euros)	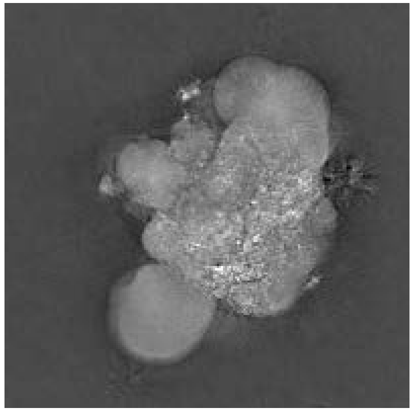	- Apoptosis<br>- Necrosis
Annexin-V (confocal or fluorescence microscopy)	at a given time	fixed cells	several conditions	available	50 tests<br><br>(247 euros)	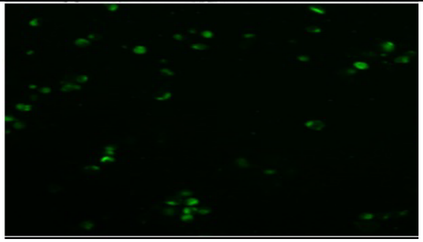	- Apoptosis
MDC (confocal or fluorescence microscopy)	at a given time	fixed cells / nonfixed cells	several conditions	available	20 tests<br><br>(68 euros)	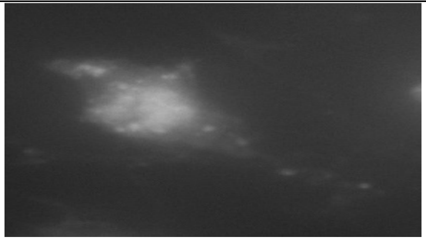	- Autophagy

Electron microscopy	at a given time	fixed cells	limited in number of conditions because as cumbersome technique requiring trained and experienced team	instrument unusual	250 euros per conditions	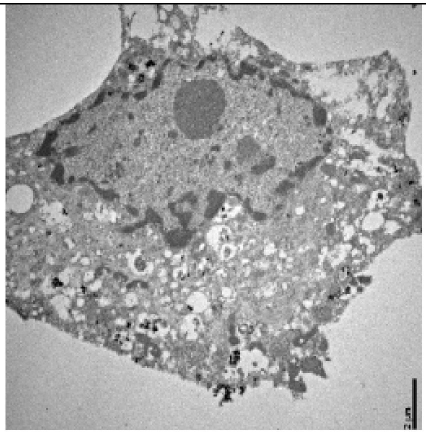	- Apoptosis<br>- Autophagy

Supplementary Video

Supplementary Video 1: Digital holograms of RA-FLS treated with CdTe-QDs 510 nm at 1 µg/mL during 27 hours.

Supplementary Video 2: Three-dimensional video of RA-FLS taken after 24-hours exposure with CdTe-QDs 10 µg/mL by confocal microscopy in fluorescence mode.

Supplementary Video 3: Time-lapse video of ten synoviocytes in the presence of CdTe-QDs, taken in images every 30 min during 48 hours.
